# Supplementary material for: Respiration and growth of Paracoccus denitrificans R-1 with nitrous oxide as an electron acceptor
Source: Microbiol Spectr. 2024 Apr 22;12(6):e03811-23. doi: 10.1128/spectrum.03811-23 (PMC11237620; doi:10.1128/spectrum.03811-23)
Supplement: Supplemental material — Fig. S1 and S2; Tables S1 to S6. Fig. S1: N2O reduction process curve of Paracoccus denitrificans R-1 under different electron donors (carbon source). Fig. S2: Effect of different concentrations of inhibitors on the N2O reduction of P. denitrificans R-1. Table S1: Growth of Paracoccus denitrificans R-1 in culture with N2O as the only electron acceptor. Table S2: Growth of Paracoccus denitrificans R-1 at different temperatures. Table S3: Growth of Paracoccus denitrificans R-1 at different pH. Table S4: Growth of Paracoccus denitrificans R-1 at different O2 concentration. Table S5: Growth of Paracoccus denitrificans R-1 with additional nitrogen sources. Table S6: N2O consumption rate of Paracoccus denitrificans R-1 in 8 h cultured with different electron donors. [file spectrum.03811-23-s0001.docx]

Supplementary materials

**Respiration and growth of** ***Paracoccus denitrificans* R-1 with nitrous oxide as an electron acceptor**

Jiaxian Zhou^1,2^, Wenfang Deng^1^, Jiapeng Wu^1^, Hua Xiang^1^, Jih-Gaw Lin^3^, Yiguo Hong^1*^

^1^Institute of Environmental Research at Greater Bay Area; Key Laboratory for Water Quality and Conservation of the Pearl River Delta, Ministry of Education, Guangzhou University, Guangzhou, 510006, China

^2^School of Environmental Science and Engineering, Guangzhou University, Guangzhou, 510006, China

^3^Institute of Environmental Engineering, National Yangming Chiao Tung University, 1001 University Road, Hsinchu City, 30010, Taiwan

*Corresponding Author: Email: yghong@gzhu.edu.cn, Phone/Fax: 020-39337914

Contents of this file

Tables 6

Figures 2

**Table S1 Growth of *Paracoccus denitrificans* R-1 in culture with N_2_O as the only electron acceptor.**

| Initial OD_600_ | Final OD_600_ | OD_600_ increase | Exponential growth rate (h^-1^) |
| --- | --- | --- | --- |
| 0.0675±0.0021 | 0.1925±0.0007 | 0.125±0.0028 | 0.0437±0.0015 |

For exponential growth rate, its calculation formula is (lnX_t_-lnX_0_)/T, where Xt is the final OD_600_, X_0_ is the initial OD_600,_ T is the culture time. Tables S2-S5 are the same.

**Table S2 Growth of *Paracoccus denitrificans* R-1 at different temperatures.**

| Temperature (℃) | Initial OD_600_ | Final OD_600_ | OD_600_ increase | Exponential growth rate (h^-1^) |
| --- | --- | --- | --- | --- |
| 20 | 0.0535±0.0163 | 0.09±0.0099 | 0.0365±0.0063 | 0.0225±0.0083 |
| 30 | 0.0635±0.0021 | 0.185±0.0042 | 0.1215±0.0064 | 0.0446±0.0023 |
| 40 | 0.061±0.0057 | 0.079±0.0177 | 0.0175±0.0233 | 0.0101±0.0133 |

**Table S3 Growth of *Paracoccus denitrificans* R-1 at different pH.**

| PH | Initial OD_600_ | Final OD_600_ | OD_600_ increase | Exponential growth rate (h^-1^) |
| --- | --- | --- | --- | --- |
| 5 | 0.0565±0.0021 | 0.089±0.0057 | 0.0325±0.0078 | 0.0189±0.0042 |
| 6 | 0.06±0.0042 | 0.0855±0.0035 | 0.255±0.0007 | 0.0148±0.0012 |
| 7 | 0.0585±0.0035 | 0.1855±0.0233 | 0.127±0.0198 | 0.0479±0.0027 |
| 8 | 0.0555±0.0021 | 0.1395±0.0629 | 0.084±0.0651 | 0.0362±0.0211 |
| 9 | 0.061±0.0028 | 0.1285±0.0474 | 0.0675±0.0445 | 0.0296±0.0138 |

**Table S4 Growth of *Paracoccus denitrificans* R-1 at different O_2_ concentration.**

| O_2_ concentration (v/v%) | Initial OD_600_ | Final OD_600_ | OD_600_ increase | Exponential growth rate (h^-1^) |
| --- | --- | --- | --- | --- |
| 0 | 0.0635±0.0021 | 0.185±0.0042 | 0.1215±0.0021 | 0.0446±0.0004 |
| 5 | 0.0645±0.0007 | 0.1335±0.0007 | 0.069±0.0001 | 0.0303±0.0002 |
| 10 | 0.0605±0.0021 | 0.118±0.0127 | 0.575±0.0106 | 0.0277±0.0030 |
| 20 | 0.071±0.0028 | 0.1315±0.0092 | 0.605±0.0064 | 0.0256±0.0013 |

**Table S5 Growth of *Paracoccus denitrificans* R-1 with additional nitrogen sources.**

| nitrogen sources | Initial OD_600_ | Final OD_600_ | OD_600_ increase | Strain growth rate (OD_600_·h^-1^) |
| --- | --- | --- | --- | --- |
| Not add | 0.0655±0.0021 | 0.184±0.0141 | 0.1185±0.012 | 0.0430±0.0019 |
| NO_3_^-^ 10 mg·L^-1^ | 0.07±0.0042 | 0.2005±0.012 | 0.1305±0.0163 | 0.0438±0.0050 |
| NO_3_^-^ 20 mg·L^-1^ | 0.0575±0.0078 | 0.136±0.0014 | 0.0785±0.0092 | 0.0360±0.0060 |
| NO_3_^-^ 50 mg·L^-1^ | 0.057±0.0028 | 0.209±0.0467 | 0.152±0.0495 | 0.0536±0.0115 |
| NO_3_^-^ 100 mg·L^-1^ | 0.0555±0.0021 | 0.2205±0.0177 | 0.165±0.198 | 0.0574±0.0049 |
| NH_4_^+^ 10 mg·L^-1^ | 0.0645±0.0021 | 0.26±0.0424 | 0.1955±0.0445 | 0.0578±0.0082 |
| NH_4_^+^ 20 mg·L^-1^ | 0.075±0.0042 | 0.2855±0.0248 | 0.2105±0.0205 | 0.0557±0.0013 |
| NH_4_^+^ 50 mg·L^-1^ | 0.075±0.0014 | 0.2545±0.0615 | 0.1795±0.0629 | 0.0503±0.0110 |
| NH_4_^+^ 100 mg·L^-1^ | 0.0785±0.0049 | 0.256±0.0509 | 0.1775±0.0559 | 0.0489±0.0110 |

**Table S6** **N_2_O consumption rate of *Paracoccus denitrificans* R-1 in 8 h cultured with different electron donors.**

| Electron donor | Initial N_2_O concentration (μmol) | N_2_O concentration after 8 h culture (μmol) | N_2_O consumption rate in 8 h (μmol·h^-1^) |
| --- | --- | --- | --- |
| Sodium formate | 101.26±0.66 | 71.72±3.89 | 3.69±0.41 |
| Sodium acetate | 104.47±3.71 | 6.22±3.277 | 12.28±0.87 |
| Sodium pyruvate | 119.56±3.92 | 94.87±1.86 | 3.09±0.72 |
| Sodium lactate | 107.66±2.98 | 14.60±0.57 | 11.63±0.44 |
| Sodium succinate | 98.38±1.62 | 68.84±0.54 | 3.69±0.13 |
| Sodium salicylate | 98.19±7.43 | 86.81±9.24 | 1.42±0.23 |
| Sodium citrate | 101.93±2.62 | 105.09±2.12 | -0.39±0.59 |
| Glucose | 104.26±3.71 | 68.54±5.39 | 4.46±0.21 |
| Glycerol | 93.88±0.38 | 80.81±4.31 | 1.63±0.49 |
| Ethanol | 94.85±3.45 | 7.87±2.65 | 10.87±0.10 |
| Sodium propionate | 88.69±3.38 | 78.06±6.36 | 1.33±0.37 |

**Figure S1**





**Fig. S1** N_2_O reduction process curve of *Paracoccus denitrificans* R-1 under different electron donors (carbon source). Eleven electron donors and the only electron acceptor N_2_O were selected to incubate *P. denitrificans* R-1. Including sodium formate (A), sodium acetate (B), sodium pyruvate (C), sodium lactate (D), sodium succinate (E), sodium salicylate (F), sodium citrate (G), glucose (H), glycerol (I), ethanol (J), sodium propionate (K), and no carbon source (L). Data points are averages of duplicate experiments and error bars represent standard deviations.

**Figure S2**





**Fig. S2** Effect of different concentrations of inhibitors on the N_2_O reduction of *P. denitrificans* R-1. Include rotenone (A) and dicoumarol (B). Data points are averages of duplicate experiments and error bars represent standard deviations.
